# Methylation by a Unique α-class N4-Cytosine Methyltransferase Is Required for DNA Transformation of *Caldicellulosiruptor bescii* DSM6725

**DOI:** 10.1371/journal.pone.0043844

**Published:** 2012-08-22

**Authors:** Daehwan Chung, Joel Farkas, Jennifer R. Huddleston, Estefania Olivar, Janet Westpheling

**Affiliations:** 1 Department of Genetics, University of Georgia, Athens, Georgia, United States of America; 2 The BioEnergy Science Center, Department of Energy, Oak Ridge National Laboratory, Oak Ridge, Tennessee, United States of America; Oregon State University, United States of America

## Abstract

Thermophilic microorganisms capable of using complex substrates offer special advantages for the conversion of lignocellulosic biomass to biofuels and bioproducts. Members of the Gram-positive bacterial genus *Caldicellulosiruptor* are anaerobic thermophiles with optimum growth temperatures between 65°C and 78°C and are the most thermophilic cellulolytic organisms known. In fact, they efficiently use biomass non-pretreated as their sole carbon source and in successive rounds of application digest 70% of total switchgrass substrate. The ability to genetically manipulate these organisms is a prerequisite to engineering them for use in conversion of these complex substrates to products of interest as well as identifying gene products critical for their ability to utilize non-pretreated biomass. Here, we report the first example of DNA transformation of a member of this genus, *C. bescii*. We show that restriction of DNA is a major barrier to transformation (in this case apparently absolute) and that methylation with an endogenous unique α-class N4-Cytosine methyltransferase is required for transformation of DNA isolated from *E. coli*. The use of modified DNA leads to the development of an efficient and reproducible method for DNA transformation and the combined frequencies of transformation and recombination allow marker replacement between non-replicating plasmids and chromosomal genes providing the basis for rapid and efficient methods of genetic manipulation.

## Introduction

Current methods for the use of lignocellulosic biomass as a substrate for microbial conversion to products of interest rely on pretreatment of the biomass with acids, alkali or organic solvents, often at high temperature [1], [2] and the addition of hydrolytic enzymes that partially digest the plant cell walls [3]. Enzymatic pretreatment is particularly expensive and often prohibitive for the production of low value commodity products from biomass. Thermophilic microorganisms offer special advantages for biomass conversion, in part, because they offer the potential to decrease hydrolysis times by several-fold with the same cellulase loading or to decrease cellulase loading by several fold at constant hydrolysis times. Organisms that can use complex biomass as substrate reduce the need for pretreatment and enzymatic hydrolysis and, therefore, the cost of the process. *Clostridium thermocellum* and *Thermoanaerobacterium saccharolyticum* are prominent examples of thermophilic (T_opt_ = 60^°^C and 55^°^C respectively) bacteria that specialize in the solubilization and fermentation of crystalline cellulose to products that include ethanol. Recent advances in the development of genetic tools that facilitate metabolic engineering of these organisms [4], [5] have made them leading candidates for consolidated bioprocessing (CBP) of biomass to biofuels. *Thermoanaerobacterium saccharolyticum* has been engineered to produce ethanol at near-theoretical yield from a variety of sugars derived from biomass pretreatment [4] and efforts to identify microbes for direct conversion of biomass is an active area of investigation [6]. *Caldicellulosiruptor* species have the ability to utilize non-pretreated biomass including both low-lignin napier and Bermuda grasses as well as high–lignin switchgrass and a hardwood, popular, for growth [7]. Members of this genus are the most thermophilic of all known organisms capable of using non-pretreated cellulosic biomass [8]. The sequences of eight *Caldicellulosiruptor* genomes have been published and reveal enzymes likely to be important in lignocellulose utilization [9]–[12]. In addition, microarray analysis of cells grown on various substrates implicates specific genes and gene clusters in biomass degradation [13], [14].

There are many interesting microorganisms that do interesting and important chemistry but the ability to manipulate them genetically is essential to making them useful. Development of genetic systems for hyperthermophiles, in general, presents many challenges, some of which result from the extreme growth requirements of these organisms. One of the most significant barriers to the genetic manipulation of uncharacterized bacteria in general and hyperthermophiles in particular is the lack of selectable markers. Antibiotic selection strategies used in mesophilic bacteria are typically ineffective in hyperthermophiles, because of the instability of either the drug or the heterologously expressed resistance protein at high temperatures [15], [16]. Nutritional markers can be particularly useful for genetic selection if an organism is able to grow on a defined medium. We developed a method for selection of transformation in *Caldicellulosiruptor bescii* based on the loss of the uracil biosynthetic enzyme orotidine-5′-monophosphate (OMP) decarboxylase (*pyrF*), first described in yeast [17] and since used successfully in both bacteria and archaea [18]–[23].

We recently discovered that *Caldicellulosiruptor bescii* has a potent restriction endonuclease, CbeI, an isoschizomer of HaeIII that cleaves unmethylated sequences at 5′-GG/CC-3′ [24]. Type II restriction endonucleases like CbeI have been shown to be a barrier to DNA transformation of several bacterial strains and overcoming restriction by the hosts was a key to successful transformation. Approaches include engineering the transforming DNA to contain fewer restriction sites [25]–[27], *in vitro* methylation by purified methyltransferases [28] or cell extracts [29], [30], or *in vivo* methylation by *E. coli* [26], [31]. We were unable to transform *C. bescii* in many attempts using a variety of transformation procedures and speculated that restriction by CbeI might be a barrier. As CbeI (Cbes 2438) recognizes and cleaves the same sequence as HaeIII, we anticipated that methylation by HaeIII methyltransferase (M.HaeIII) might protect DNA from *E. coli* from cleavage by CbeI and eliminate it as a barrier to DNA transformation. M.HaeIII (NEB) partially protected DNA from cleavage by both HaeIII and CbeI *in vitro*, but no transformants were detected when this DNA was used in a variety of transformation protocols. DNA isolated from *E. coli* strains containing combinations of methyltransferases that facilitated transformation of the thermophiles *Bacillus methanolicus* [26] and *Clostridium thermocellum* [32] also failed to transform *C. bescii* using the same protocols. A gene for an apparent cognate methyltransferase, M.CbeI (Cbes 2437) is present adjacent to CbeI in the *C. bescii* genome as well as the genomes of *C. hydrothermalis* 108 (Calhy 0409) and *C. kristjanssonii* 177R1B (Calkr 2088) and is second only to dam-like methyltransferase (methylates 5′-GATC-3′), which is present in all sequenced *Caldicellulosiruptor* isolates in its occurrence [9]. Cytosine methyltransferases methylate cytosine to either 5-methylcytosine (m5C), as for M.HaeIII, [33] or more rarely to N4-methylcytosine (m4C). A study of the methylation patterns of DNA in thermophiles with optimal growth temperatures above 60°C, revealed that methylation to m4C is actually more common than m5C, perhaps because m5C is more readily deaminated to thymine by heat [34].

Here we show that restriction is an important, perhaps absolute barrier, to transformation of *Caldicellulosiruptor* by DNA from *E. coli* and that methylation by a novel α-class Type II cytosine methyltransferase [35] overcomes this barrier. While the apparent transformation frequency is low, the combined frequencies of transformation and recombination allow maker replacement of chromosomal genes with non-replicating vectors providing an essential tool to generate deletions to identify genes important for biomass utilization, gene substitutions, His-tags for protein purification, and expression of heterologous proteins to extend substrate utilization and biomass conversion in these organisms.

## Results

### A spontaneous deletion of the *C. bescii pyrBCF* locus allows nutritional selection of transformants

**Figure 1:**
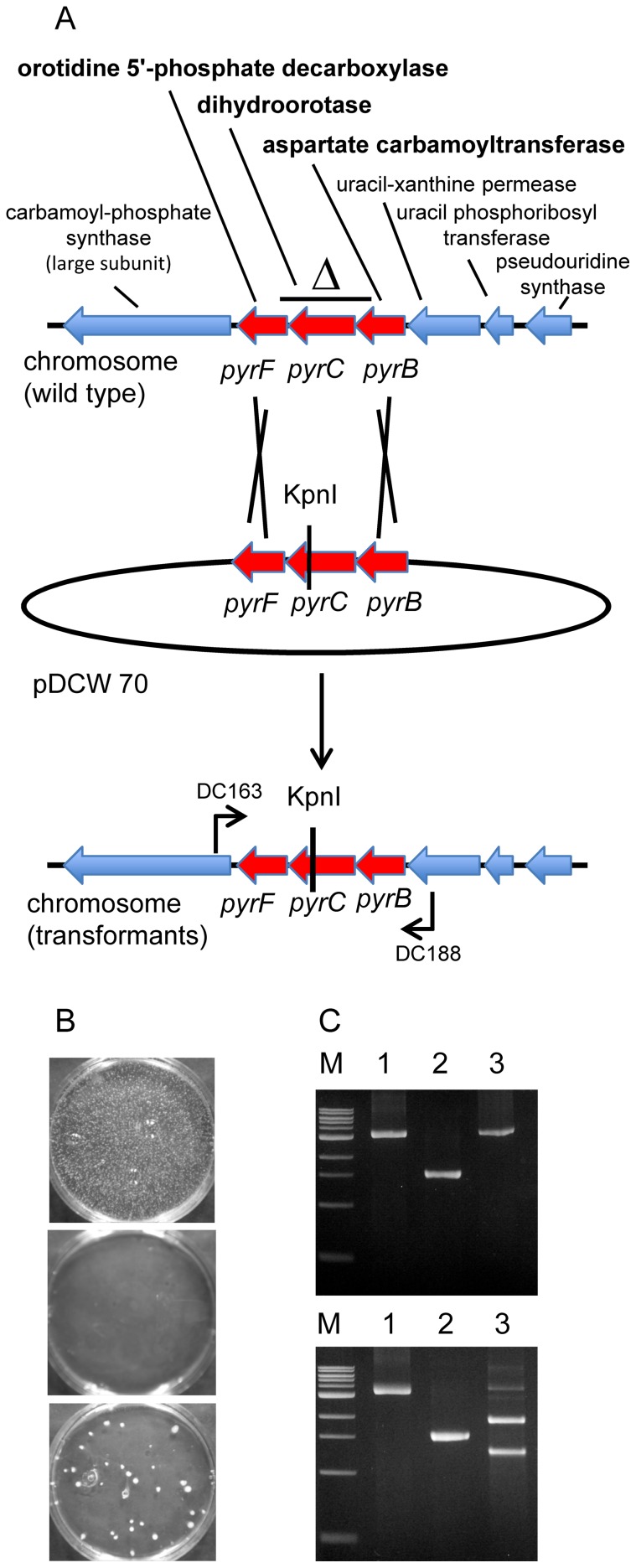
Confirmation of transformation and marker replacement in the Δ*pyrBCF* strain. (A) A diagram of the *pyrBCF* locus in the *C. bescii* chromosome. The line above the diagram depicts the extent of the spontaneous deletion in the Δ*pyrBCF* strain. pDCW70 contains the wild type *pyrBCF* alleles with an engineered KpnI site used to select marker replacement of the deletion depicted with primers used to confirm the structure of the chromosome in the transformant. B) Δ*pyrBCF* electrocompetent cells after electro-pulse with no DNA added plated onto defined medium + uracil (top plate), Δ*pyrBCF* electrocompetent cells after electro pulse with unmethylated pDCW70 DNA plated onto defined medium w/o uracil (middle plate), Δ*pyrBCF* electrocompetent cells after electro-pulse with M.CbeI methylated pDCW70 DNA plated onto defined medium w/o uracil (bottom plate). (C) PCR products amplified using primers DC163 and DC188 (upper gel) digested with KpnI (lower gel). M: 1kb DNA Ladder (NEB). lane 1: amplified from wild type cells (3.2 kb); lane 2: amplified from Δ*pyrBCF* (1.63 kb); lane 3: amplified from transformants (1.9 and 1.3 kb cleavage products).

Attempts to use a thermostable kanamycin resistance gene previously used for selection of transformants in *Thermoanaerobacterium* species at 60°C [36], [37] to select transformants in *C. bescii* was complicated by the fact that *C. bescii* (that grows optimally at 75°C) grows very poorly at or below 70°C. In fact, growth at 60°C increased the spontaneous mutation frequency significantly, from 10^−7^ to 10^−5^, making the detection of transformants over this background of spontaneous drug resistance problematic. Attempts to use a hygromycin phosphotransferase (*hph*) gene from *E. coli*, that had been selected for function at 85°C in *Sulfolobus solfataricus* [38] were compromised by the level of natural resistance to hygromycin in *C. bescii* and the fact that, in our hands, this hygromycin resistance gene is not reliable for selection above 70°C. To generate a mutant strain for nutritional selection of transformants, *C. bescii* cells were plated on 5-fluoroorotic acid (5-FOA). OMP decarboxylase, encoded by the *pyrF* gene in bacteria (*ura3* in yeast), converts the pyrimidine analog 5-fluoroorotodine monophosphate to 5-fluorouridine monophosphate which is ultimately converted to fluorodeoxyuridine by the uracil biosynthetic pathway, a toxic product that kills growing cells that are synthesizing uracil [17]. Mutants of *pyrF* are, therefore, uracil auxotrophs resistant to 5-FOA. Spontaneous resistance to 5-FOA (8 mM) was observed at a frequency of approximately 10^−5^ at 60°C. One such mutant contained a deletion that included part of the carboxy terminus of *pyrF* (Cbes1377) open reading frame, the entire *pyrC* (Cbes1376) open reading frame and the amino terminus of *pyrB* (Cbes1375) open reading frame, diagrammed in Fig 1A, and was used for further analysis. The extent of the deletion was defined by PCR amplification of the *pyrBCF* region in the mutant (Fig 1C) and subsequent sequencing of the PCR product. Since mutations in *pyrE* also lead to uracil auxotrophy and 5FOA resistance, the region around the *pyrE* locus (Cbes1382) was amplified from this strain and sequenced to ensure that it was wild type. *pyrE* is located 3 ORFs and 2.4 kb downstream of *pyrA* and 5.6 kb downstream of *pyrF* and is unlikely to be transcriptionally coupled to *pyrBCF* or *pyrA*. While the deletion would be expected to affect only the *pyrBCF* genes, qPCR analysis was performed to monitor expression of the *pyrA* gene (Cbes1378) as well as the Cbes1374 open reading frame predicted to encode a uracil xanthine permease. Expression of *pyrA* and Cbes1374 in the deletion mutant was indistinguishable from the wild type suggesting that the deletion within the *pyrBCF* locus did not affect expression of surrounding genes. The Δ*pyrBCF* strain, JWCB002, (Table 1) was a tight uracil auxotroph and because it contained a deletion, reversion to uracil prototrophy was not a concern making prototrophic selection possible no matter how low the frequency of transformation. Growth of this mutant supplemented with uracil (20 µM) was indistinguishable from that of the wild type, reaching a cell density of ∼2×10^8^ in 20 hours. To assay transformation, a non-replicating plasmid was constructed with the wild type copy of the *pyrBCF* locus but containing an engineered restriction site within the *pyrC* open reading frame to distinguish it from the chromosomal wild type allele. This plasmid was used to transform the *pyrBCF* deletion strain selecting marker replacement events that repaired the deletion (strategy diagrammed in Fig 1A).

**Table 1:** Strains/plasmids used and constructed in this study.

Strains/Plasmids	Description	Source
DSM 6725	*Caldicellulosiruptor bescii* wild type	DSMZ^1^
JWCB002	*C. bescii* Δ*pyrBCF*	This study
JWCB003	JWCB002 with *pyrBCF* restored by marker replacement	This study
JW 284	*E. coli* DH5α containing pDCW73	This study
pDCW 70	Contains the *pyrBCF* locus with an engineered KpnI site	This study
pDCW 73	M.CbeI Expression vector	This study

1 German Collection of Microorganisms and Cell Cultures.

We were unable to transform *C. bescii* in many attempts using this strategy with DNA isolated from *E. coli*. We used and modified methods known to work well for other Gram-positive bacteria including electroporation [36], [39], [40], artificially induced competence [41], natural competence [19], [42], and methods that altered membrane permeability [43]. Mating with *E. coli*, a method of DNA transfer that works well for similar bacteria [15], [44], did not work for *C. bescii* or the other *Caldicellulosiruptor* species we tested using the same approach.

### *In vivo* and/or *in vitro* methylation of DNA from *E. coli* partially protects DNA from cleavage but does not allow transformation of *C. bescii*

**Figure 2:**
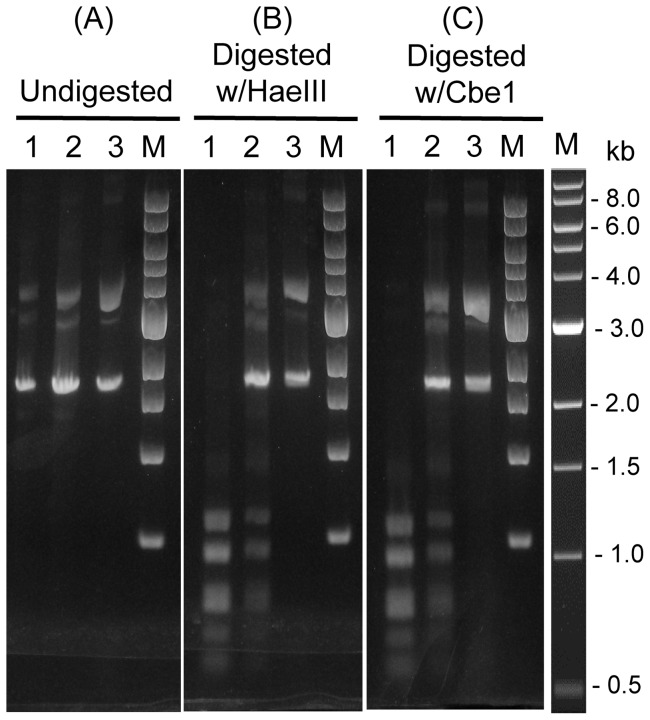
Protection of DNA by M.CbeI or M.HaeIII from digestion *in vitro*. In each panel, lane 1 is unmethylated plasmid DNA (pUC18) isolated from *E. coli* (*dam^+^dcm^+^*), lane 2 is plasmid DNA methylated *in vitro* with M.HaeIII (NEB) at 37°C, lane 3 is plasmid methylated with purified M.CbeI at 78°C. (A) undigested. (B) digested with HaeIII (C) digested with purified CbeI. DNAs were subjected to electrophoresis in a 1.2% TAE-agarose gel, and then stained with ethidium bromide. M: 1kb DNA ladder (NEB).

CbeI, a potent restriction endonuclease in *C. bescii*, recognizes and cleaves the same sequence as HaeIII, unmethylated DNA at the sequence 5′-GG/CC-3′ [24]. We anticipated that methylation by HaeIII methyltransferase (M.HaeIII) might protect DNA from *E. coli* from cleavage by CbeI and overcome it as a potential barrier to DNA transformation. This, in fact, was shown to be true for transformation of the archaeon *Sulfolobus acidocaldarius* using M.HaeIII to avoid restriction by SuaI [45]. Plasmid DNA treated with M.HaeIII, *in vitro*, was partially protected from cleavage by both HaeIII and CbeI *in vitro* (Fig 2), but no transformants were obtained when this DNA was used in electroporation experiments or added to cells that had been subjected to a procedure to induce natural competence in *Mycobacterium* and *Thermoanaerobaterium* species [41], [42]. In addition, various strains of *E. coli* containing combinations of methyltranferases were used to prepare DNA for transformation (Table 2), a method that was successful for transforming *Clostridium thermocellum* using a *dam^+^dcm^−^ E. coli* strain [32]. No transformants of *C. bescii* were detected using DNA from these strains. In total we performed more than 1000 electroporation experiments varying conditions for cell growth, transformation conditions, and assay conditions as well as using DNA from different strains of *E. coli* or methylated with M.HaeIII.

**Table 2:** Influence of methylation on transformation efficiency of pDCW70.

*E. coli* strain used to prepare pDCW70	Transformation efficiency (Transformants/µg of DNA)^a^
DH5α (*dam^+^dcm^+^*)	ND^b^
BL21 (*dam^+^dcm^−^)*	ND^b^
ET12567 (*dam^−^dcm^−^*)	ND^b^
DH5α (*dam^+^dcm^+^)/*M.HaeIII	ND^b^
DH5α (*dam^+^dcm^+^)/*M.CbeI	∼50^c^

a Each transformation experiment used approximately 10^9^ cells and 600 ng of transforming DNA.

b ND (Not detected) : based on at least 30 independent transformation experiments.

C Average of the results of five independent transformation experiments.

### M.CbeI is a novel α-class N4-Cytosine methyltransferase

**Figure 3:**
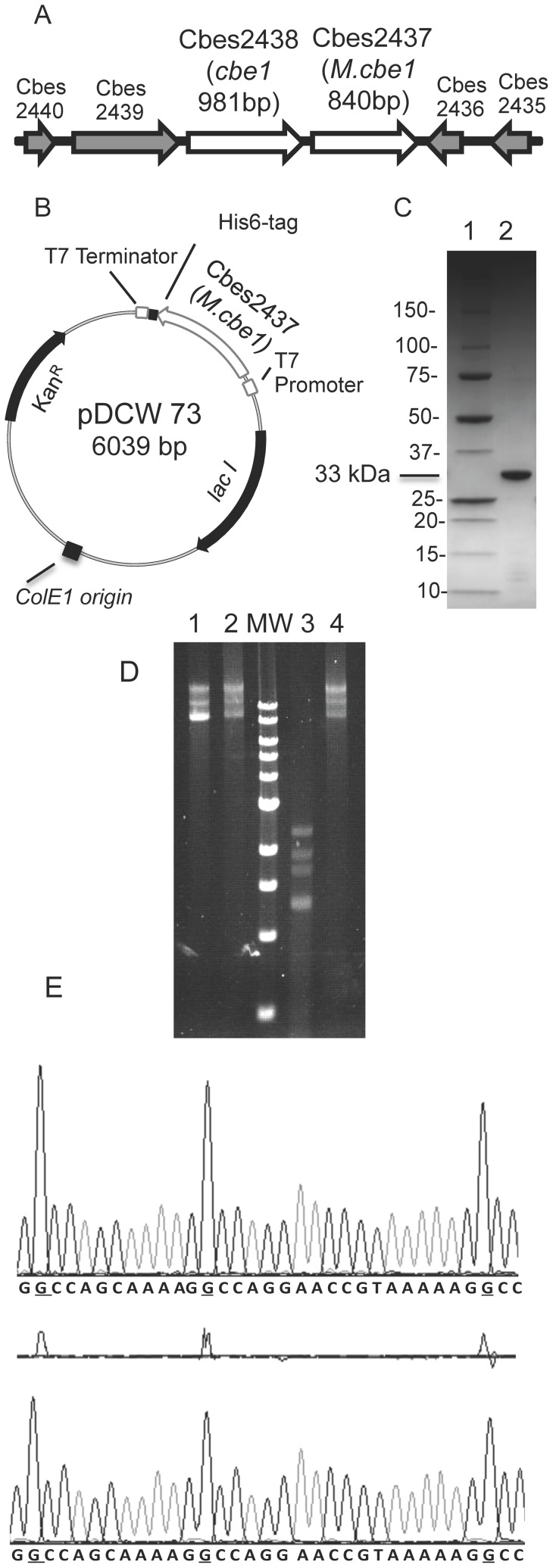
Cloning, expression, purification and partial characterization of M.CbeI. (A) The region of the *C. bescii* chromosome containing CbeI and M.CbeI. (B) Diagram of pDCW73 used to produce a His-tagged version of M.CbeI in *E. coli*. (C) Purified M.CbeI displayed on a 10–20% Tris-HCl gradient gel (CriterionTM Precast Gel, Bio-Rad Laboratories, Hercules, CA) stained with coomassie blue. lane 1: protein molecular weight standards (BioRad); lane 2: 15 ng of purified M.CbeI protein. (D) lane 1, undigested unmethylated pDCW70; lane 2, undigested M.CbeI methylated pDCW70; lane 3, unmethylated pDCW70 digested with purified CbeI; lane 4, M.CbeI methylated pDCW70 digested with purified CbeI, MW: 1 kb DNA ladder (NEB). (E) DNA sequence traces of DNA methylated with M.CbeI (top panel) or M.HaeIII (bottom panel). Differences in the G residue signals between M.HaeIII and M.CbeI pUC18 are shown in the middle panel.

As shown in Fig 3A, the region of the chromosome that contains CbeI also contains an open reading frame, Cbes2437, predicted to encode an adenine specific methyltransferase [46]. This open reading frame was cloned into an *E. coli* expression vector, pDCW73 (Fig 3B) that placed a His-Tag at the carboxy terminus of the protein allowing purification on a Ni-NTA column. *E. coli* cells containing this plasmid were viable at 23°C but not 37°C suggesting that expression of M.CbeI was toxic to growing cells. Expression of this methyltransferase was, therefore, performed at 23°C to avoid problems related to toxicity and in *E. coli* BL21-CodonPlus(DE3)-RIPL to alleviate problems arising from the significant differences in codon usage between M.CbeI and *E. coli*. Purified M.CbeI from *E. coli* was the size predicted from the open reading frame, 33 kDa (Fig 3C). No cleavage of DNA was detected by purified CbeI at 75°C when DNA from *E. coli* was methylated *in vitro* by the purified methyltransferase (Fig 3D) and we named this enzyme M.CbeI. To determine the optimal temperature for M.CbeI methyltransferase activity, we performed the *in vitro* methylation reactions with purified M.CbeI at temperatures ranging from 25°C to 100°C and tested the modified DNA for restriction by CbeI. Reactions performed between 65°C and 85°C, the growth temperature range of *C. bescii*, resulted in the best protection against cleavage by CbeI.

Even though CbeI is an isoschizomer of HaeIII and M.CbeI would be expected to methylate the same sequence as M.HaeIII, methyltransferases vary in the sites of methylation and specific or cognate methylation may be required for full protection. The pattern of DNA methylation by M.CbeI was compared to that by M.HaeIII using a method [47], [48] that relies on the fact that the extent of incorporation of fluorescently labeled dideoxynucleotides during DNA sequencing is influenced by methylated bases in the template DNA. pUC18 DNA was methylated *in vitro* by either M.CbeI or M.HaeIII and direct sequencing of the DNA revealed that DNA methylated with M.CbeI showed a higher degree of incorporation of dideoxyguanosine in the 5′-G**G**CC-3′ recognition sequence than DNA methylated with M.HaeIII. N4-methylcytosine results in an increase in the complementary G (G**G**CC) signal and this signature (Fig 3E) indicates that M.CbeI methylated DNA contains N4-methylcytosine (m4C). M.HaeIII methylates the C5 position of cytosine (m5C).

### Methylation of *E. coli* DNA, *in vitro*, with purified M.CbeI protein allows transformation of *C. bescii*

Plasmid DNA from *E. coli* (*dam^+^dcm^+^*) methylated by M.CbeI *in vitro* readily transformed the *C. bescii ΔpyrBCF* strain resulting in marker replacement of the deletion with the wild type allele containing the engineered KpnI site (Fig 1). Amplification of the *pyrBCF* region from wild type *C. bescii* resulted in a 3.2 kb product while the product generated from the deletion strain was 1.63 kb. Amplification of this region in the transformant generated a wild type size product. Digestion with KpnI resulted in no cleavage of the product generated from the wild type or the Δ*pyrBCF* mutant. The product generated from transformant was digested with KpnI showing that the transformant contained the allele from the plasmid and its presence in the *C. bescii* chromosome resulted from marker replacement (Fig 1C). Transformation efficiencies were routinely on the order of 50 transformants per microgram of non-replicating plasmid DNA (Table 2, Fig 1 B). This extremely low transformation efficiency may be an underestimate of the actual efficiency as the plating efficiency of *C. bescii* on selective solid medium is less than 10^−4^ (plating 10^6^ cells as determined by cell count resulted in fewer than 100 colonies).

## Discussion

While there are many challenges in the development of transformation protocols, restriction of DNA from *E. coli* by host bacteria is often an issue. Restriction/modification of DNA, first recognized as a mechanism of protection against phage infection [49], varies in effectiveness depending on the activity of restriction endonuclease and the methylation state of the DNA substrate. Methylation of DNA may either facilitate or limit the activity of endonucleases and plays a major role in transformation of heterologous DNA no matter what the source of the DNA or the host for transformation. Transformation of DNA from *E. coli* to *Caldicellulosiruptor bescii* is apparently especially sensitive to restriction/modification and here we show that the use of a novel endogenous methyltransferase provided specific modification of DNA from *E. coli* that allowed efficient transformation.

**Figure 4:**
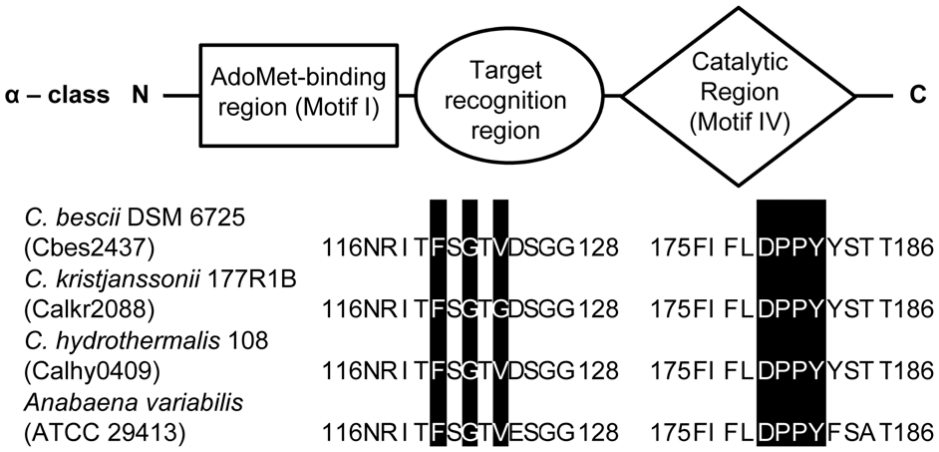
Domain structure of M.CbeI. Predicted functional domains of M.CbeI and sequence alignments of conserved motifs of three M.CbeI homologues from members of *Caldicellulosiruptor* species as well as DmtB from *Anabaena variabilis*, which also contains an M.CbeI homologue showing unique features of this methyltransferase.

M.CbeI was annotated as a D12 class N6 adenine-specific DNA methyltransferase in GenBank [46], but our analysis clearly shows that it functions as a cytosine specific methyltransferase. Like all known methyltransferases it contains a conserved F_G_G amino acid motif that facilitates interaction with S-adenosylmethionine, the source of the methyl group in these reactions. M.CbeI also contains a DPPY motif typical of N6-adenine methyltransferases, all of which contain a (D/N)PP(Y/F) motif [50], [51]. Its SPP(Y/F) motif is the hallmark of N4 cytosine methyltransferases active site [52], making M.CbeI unusual in that it contains a DPPY motif in the active site (Fig 4). Furthermore, the M.CbeI protein has no reported significant sequence similarity to any characterized N4 cytosine methyltransferase. Our own search revealed similarity to DmtB from *Anabaena variabilis* ATCC 29413 (Fig 4), which has been shown to have m4C methyltransferase activity specific to the inner cytosine in the 5′-GG**C**C-3′ recognition sequence [53]. These proteins, which show 57% amino acid identity, represent a new α-class methyltransferase specific for the GGCC sequence, different from the previously characterized β-class of N4-methyltransferases in hyperthermophiles, M.SuaI [45] and M.PhoI [54], isolated from the archaea *Sulfolobus acidocaldarius* and *Pyrococcus horikoshii* OT3, respectively. These data suggest that these proteins may have evolved from different common ancestors with other β-class N4-methyltransferases specific for 5′-GG**C**C-3′. M.CbeI is the first characterized α-class m4C methyltransferase from a hyperthermophile and homologs exist in two other *Caldicellulosiruptor* species, Calhy0409 (88% of protein sequence identity) from *C. hydrothermalis* 108 and Calkr2088 (85% of protein sequence identity) from *C. kristjanssonii* 177R1B (Fig 4).

One reason for the fact that even though M.HaeIII partially protects DNA from cleavage by CbeI, it is not sufficient to allow transformation of *C. bescii* is the activity of CbeI itself. In a previous study [45], M.HaeIII-modified DNA (m5C) was cleaved at reasonable efficiency by purified SuaI, a GGCC specific restriction enzyme completely blocked by m4C methylation at the inner cytosine residue in high concentrations. M.HaeIII is also known to have a significant level of promiscuous methylation activity at non-canonical sites [55] and may actually increase restriction activity in vector DNA by methyl-directed restriction enzymes.

Efforts to optimize the transformation procedure for *C. bescii* have included adding cell wall weakening agents (isoniacin or glycine) during cell growth, altering temperature during the preparation of electro-competent cells, changing the composition of the washing and electroporation buffers, altering incubation times and temperatures of the cells with DNA prior to electric-pulse, varying the electrical settings during the electric pulse, and altering the composition of the recovery medium and incubation period before plating onto selective medium. None of these modifications have improved the efficiency but as optimization of these procedures is largely empirical we continue to investigate the possibilities. Attempts to generalize the use of MCbeI to facilitate transformation of other *Caldicellulosiruptor* species are also in progress.

## Methods and Materials

### Strains and growth conditions

All strains used in this study are listed in Table 1. All growth of *Caldicellulosiruptor* species was preformed under anaerobic conditions in in modified DSMZ 516 medium [24] at a final pH 6.8. Liquid cultures were inoculated with a 1–2% inoculum or with a single colony and then incubated at 75°C overnight in anaerobic culture bottles or Hungate tubes degassed with at least three cycles of vacuum and argon. A solid medium was prepared by mixing an equal volume of liquid medium at a 2X concentration with 1% (wt/vol) Phytagel (Sigma) previously autoclaved to solubilize. Both solutions were maintained at 95°C and poured into petri dishes immediately after mixing. Initial plating of *C. bescii* in soft agar overlays allowed the cells to grow but they did not form discrete colonies because of the soft and liquid nature of the agar matrix. Increasing the agar concentration from 0.3% to 1.5% in the overlay allowed both abundant growth and the isolation of discrete colonies. Cells from overnight cultures were pelleted, washed in 1X base salts [24] three times and resuspended in 300 – 500 µl of 1x base salts. Cell suspension (100 μl) was mixed with 4 ml of soft top agar (1.5%) and poured across the top of a solid medium plates were incubated in anaerobic jars degassed with at least three cycles of vacuum and argon at 75°C for 3 to 5 days. *E. coli* strains, DH5α (*dam^+^dcm^+^*), BL21 (*dam^+^dcm^−^*), or ET12567 (*dam^−^ dcm^−^*) were used to prepare pDCW70 DNA. Cells were grown in L broth supplemented with apramycin (50 µg/ml) and plasmid DNA was isolated using a Qiagen Mini-prep Kit. Chromosomal DNA from *C. bescii* DSM 6725 was extracted using the Quick-gDNA™ MiniPrep (Zymo) according to the manufacturer's instructions.

### Isolation and characterization of 5-FOA resistant/uracil auxotrophic mutants

*C. bescii* DSM 6725 was inoculated into 10 ml of modified DSMZ 516 medium and grown anaerobically at 60°C for 24 h. Cells were harvested at 18,000 ×g for 5 min, washed twice with 1 x base salts [24], resuspended in 1 ml of 1 x base salts and plated by mixing 100 µl of cell suspension with 4 ml of 0.3% agar and overlaying onto defined modified DSMZ 516 medium (no yeast extract or casein) supplemented with 20 µM uracil and 8 mM 5-FOA (US Biologicals, Swampscott, MA). The plates were incubated anaerobically at 60°C for three days and 5-FOA resistant colonies were transferred to 10 ml of defined modified DSMZ 516 medium with 20 µM uracil and 8 mM 5-FOA and incubated overnight at 75°C anaerobically. To test for uracil auxotrophy, cells were subcultured in defined modified DSMZ 516 medium with or without uracil (20 µM). Cell number was measured in a Petroff Houser counting chamber using a phase-contrast microscope with 40× magnification.

### Plasmid Construction and DNA manipulation

Primers used in these constructions are listed in Table 3. All PCR amplifications were performed using *Pfu* Turbo DNA polymerase (Agilent Technologies). A 1.858 kb fragment containing the pSC101 replication origin was amplified from pDCW68 [24] using primers DC081 and DC230, which contain KpnI and AatII sites at each 5′end, respectively. A 4.343 kb fragment containing the apramycin resistance and *pyrBCF* cassettes was amplified from pDCW68 using primers DC084 and DC232 to which an AatII and KpnI site had been added to each 5′ end. An additional fragment (1.801 kb) containing DNA sequences used in other work not relevant to the experiments described here was amplified using primers DC212 and DC213. These three DNA fragments were cut by restriction enzymes, KpnI and AatII, and then ligated to yield pDCW69 (8.014 kb). pDCW70 was constructed by introducing a single nucleotide change (an A to C transversion resulting in a silent mutation) in the +978 amino acid of *pyrC* (Cbes 1376) ORF using “PCR based Site Directed Mutagenesis”, using DC 214 and DC 215 primers, to create the KpnI site (GGTAC/C), in pDCW 69. To construct pDCW73, the 0.837 kb M.CbeI (Cbes 2437) open reading frame was amplified by PCR using primers DC238 and DC239 using *C. bescii* genomic DNA as template. The PCR product was digested with BamHI and XhoI and ligated to pET24d [56], which had also been digested with BamHI and XhoI. This vector contains a His-tag sequence that is added to the C-terminus of the expressed protein. All plasmids used in this study were sequenced to confirm their structure**.**

**Table 3:** Primers used in this study.

Primers	Sequences (5′ to 3′)
DC081 reverse	ACCAGCCTAACTTCGATCATTGGA
DC084 forward	TCTGACGCTCAGTGGAACGAA
DC156 forward	TTAAGAGATTGCTGCGTTGATA
DC163 forward	TCCTGAACCAATAACCAAAACCT
DC188 reverse	TTGAAACATTTGCTTGGGCTAAGA
DC212 reverse	ACCCTCAAATATAACACAAAAATTGTCCAC
DC213 forward	GTTATTATTCTCTGTGGATAAGTC
DC214 forward	AGCGGTACCATTGGGTTTGAGAC
DC215 reverse	TGC AGCAAGGTTAAATTCGACATT
DC230 forward	TCATCTGTGCATATGGACAG
DC232 reverse	TAAGAGATTGCTGCGTTGATA
DC238 forward	AGAGGATCCATGCTCAAAAACGTTCTTCGATAC
DC239 reverse	TCTCCTCGAGCAGACCAAGTGCGTATTTTTC
DC326 forward	TCAGGTCCTGCTATAAAGCCAA
DC329 reverse	AGGTGTTTGAGAGATTTCCAAGG
M13F(-20) forward	GTAAAACGACGGCCAGT
M13R(−20) reverse	GCGGATAACAATTTCACACAGG
MD003C forward	ATCTTGAAAATCAGCAGAAGAAGG
MD004C reverse	CAAAAGGATACTCAAACGCTTACC
MD005C forward	TTATAGCCCCAACAGGTAAAACC
MD006C reverse	TTACCGATATTCACTGTCATTTGC
MD007C forward	TACAACATCTTTTCCTGAGACTGC
MD008C reverse	GAACAATTGAGAGGTTGAAGATAGC
MD009C forward	TTAAAGTAAACAGAGCCAAACACG
MD010C reverse	TGGTCCTGTTGTAATGATTATTGG
MD011C forward	CAGAGCGAGTTTAAGTTCATTGC
MD012C reverse	TGATGTTGTTGTATCAGCATAAAGC
MD015C forward	ATAATCAAACTCAATTCCCTGACC
MD016C reverse	GAGAAAAAGCCTAAACATAACATCG

### Purification of His-tagged M.CbeI and *in vitro* methylation of DNA

Purification of M.CbeI was similar to the method described by Chung *et* *al.* [24]. BL21-CodonPlus(DE3)-RILP cells (Agilent Technologies), containing pDCW73, were used for M.CbeI protein expression. Cells were grown at 23^°^C in L broth supplemented with kanamycin (25 µg/ml) and chloramphenicol (50 µg/ml) to OD_600_ 0.7 and induced by addition of 0.5 mM isopropyl β-D-1-thiogalactopyranoside (IPTG) at 23°C overnight. His-tagged (carboxy terminus) M.CbeI was purified as described previously [24] except for the use of a His-Spin Protein Miniprep™ (Zymo Research). Protein concentration was determined by the Bio-Rad protein assay using bovine serum albumin (BSA) as the standard. Purified protein was displayed using sodium dodecyl sulfate (SDS) polyacrylamide gel electrophoresis (PAGE) and stained with Coomassie brilliant blue G-250 as described [57]. Protein purity was determined to be >98%.

For in *vitro* methylation, DNA isolated from *E. coli* DH5α (*dam^+^dcm^+^*) was treated with either M.CbeI or M.HaeIII methyltransferase (NEB). 5 microgram of purified M.CbeI was incubated with 50 mM Tris-HCl, 50 mM NaCl, 80 μM S-adenosylmethionine (SAM), 10 mM Dithiothreitol (DTT) at pH 8.5 and 20 µg of DNA substrate in 400 µl reaction, and incubate for 2 hours at 78°C. The M.HaeIII methylation reaction was performed according supplier's instructions. To allow complete methylation, an additional 10 units of M.HaeIII and 80 µM SAM was added to the reaction every 4 h of incubation at 37°C for a total of 12 h. Methylated DNAs were purified and concentrated by Phenol/Chloroform extraction and ethanol precipitation. The extent of protection was determined by cleavage using HaeIII and NotI (NEB) restriction enzymes according to the supplier's instructions.

### Analysis of methylation by M.CbeI

To identify the site of methylation by M.CbeI, unmodified DNA was compared to that after methylation and the changes were determined by direct visualization in automated DNA sequencing chromatograms [47], [48]. *In vitro* methylation of pUC18 DNA isolated from *E. coli* DH5α (*dam^+^dcm^+^*) was carried out using M.HaeIII (NEB) and purified M.CbeI. The efficiency of methylation was determined by cleavage of the methylated and unmethylated DNA with HaeIII (NEB), purified CbeI, and *C. bescii* cell free extracts (CFE). Digested DNA was displayed by agarose gel electrophoresis and visualization using ethidium bromide staining. Automatic sequencing was performed using primers M13F(−20) and M13R(−20) in an ABI automated PRISM big-dye-terminator system (Macrogen, Inc, Maryland). Sequences were analyzed using the Chromas Lite v2.01 (Technelysium Pty Ltd.) and ABI chromatograms were compared by aligning the Sequencing traces and using SeqDoc [58].

### Transformation of *C. bescii*

To prepare cells for transformation, 2.5 milliliter of a freshly grown JWCB002 (Δ*pyrBCF*) culture was inoculated into 500 ml of fresh medium, and incubated at 78°C to mid-log phase (OD_680_ – 0.1, approximately 10^8^ cells/ml). The cultures were cooled to room temperature for 1 hour, harvested by centrifugation (5000 × g, 15 min) at 25°C and washed twice with 250 ml of pre-chilled 10% sucrose. After the final wash, the cell pellets were resuspended in a total volume of 1 ml of pre-chilled 10% sucrose and frozen in microcentrifuge tubes in a dry ice/ethanol bath in 50 μl aliquots for storage at −80°C. Plasmid DNA (0.5–1.0 µg) was added to cells, gently mixed and incubated in 10% sucrose for 15 minutes at room temperature. Electrotransformation of the cell/DNA mixture was performed via single electric pulse (1.8 kV, 600Ω, and 25 microF) in a pre-chilled 1 mm cuvette using a Bio-Rad gene Pulser. After pulsing, cells were incubated overnight at 75°C in 10 ml modified DSMZ 516 medium supplemented with 20µM of uracil, harvested by centrifugation (at 5000×g for 20min) and resuspended in 1 ml of 1 x base salts. A cell suspension (100 microliters) was plated onto defined medium without uracil. Plates were incubated in anaerobic jars at 75°C for three to four days. To confirm marker replacement of the *pyrBCF* region in the transformants, DNA from uracil prototrophic transformants was used to amplify the chromosomal region using primers DC163 and DC188 which anneal outside the regions of the *pyrBCF* fragment contained on pDCW70 (Fig 1). PCR products of this locus amplified from the wild type, the deletion mutant and the transformants were digested with KpnI and sequenced.

#### RNA extraction and RT-qPCR analyses

Total RNA was extracted using an RNeasy Mini kit (Qiagen) and stored at −80°C. RNA was treated with RNase-free DNase (Qagen) according to manufacturer's instructions. cDNA was then prepared using the AffinityScript quantitative PCR (qPCR) cDNA synthesis kit (Agilent Technologies). All quantitative reverse transcription-PCR (RT-qPCR) experiments were carried out with an Mx3000P instrument (Stratagene) with the Brilliant SYBR green qPCR master mix (Agilent Technologies). The gene encoding pyruvate ferredoxin oxidoreductase (Cbes 0877) was used as an internal control for RNA. The primers used in RT-qPCR experiments are listed in Table 3.

## References

[1] Zhang YH, Ding SY, Mielenz JR, Cui JB, Elander RT, et al (2007) Fractionating recalcitrant lignocellulose at modest reaction conditions. Biotechnol Bioeng 97: 214–223. 17318910 10.1002/bit.21386

[2] Wyman CE (2007) What is (and is not) vital to advancing cellulosic ethanol. Trends Biotechnol 25: 153–157. 17320227 10.1016/j.tibtech.2007.02.009

[3] Negro MJ, Manzanares P, Ballesteros I, Oliva JM, Cabanas A, et al (2003) Hydrothermal pretreatment conditions to enhance ethanol production from poplar biomass. Appl Biochem Biotechnol 105 –108: 87–100. 10.1385/abab:105:1-3:87 12721477

[4] Shaw AJ, Podkaminer KK, Desai SG, Bardsley JS, Rogers SR, et al (2008) Metabolic engineering of a thermophilic bacterium to produce ethanol at high yield. Proc Natl Acad Sci U S A 105: 13769–13774. 18779592 10.1073/pnas.0801266105 PMC2544529

[5] Argyros DA, Tripathi SA, Barrett TF, Rogers SR, Feinberg LF, et al (2011) High ethanol titers from cellulose by using metabolically engineered thermophilic, anaerobic microbes. Appl Environ Microbiol 77: 8288–8294. 21965408 10.1128/AEM.00646-11 PMC3233045

[6] Lynd LR, van Zyl WH, McBride JE, Laser M (2005) Consolidated bioprocessing of cellulosic biomass: an update. Curr Opin Biotechnol 16: 577–583. 16154338 10.1016/j.copbio.2005.08.009

[7] Yang SJ, Kataeva I, Hamilton-Brehm SD, Engle NL, Tschaplinski TJ, et al (2009) Efficient degradation of lignocellulosic plant biomass, without pretreatment, by the thermophilic anaerobe “Anaerocellum thermophilum” DSM 6725. Appl Environ Microbiol 75: 4762–4769. 19465524 10.1128/AEM.00236-09 PMC2708433

[8] Blumer-Schuette SE, Kataeva I, Westpheling J, Adams MW, Kelly RM (2008) Extremely thermophilic microorganisms for biomass conversion: status and prospects. Curr Opin Biotechnol 19: 210–217. 18524567 10.1016/j.copbio.2008.04.007

[9] Blumer-Schuette SE, Ozdemir I, Mistry D, Lucas S, Lapidus A, et al (2011) Complete genome sequences for the anaerobic, extremely thermophilic plant biomass-degrading bacteria Caldicellulosiruptor hydrothermalis, Caldicellulosiruptor kristjanssonii, Caldicellulosiruptor kronotskyensis, Caldicellulosiruptor owensensis, and Caldicellulosiruptor lactoaceticus. J Bacteriol 193: 1483–1484. 21216991 10.1128/JB.01515-10 PMC3067630

[10] Elkins JG, Lochner A, Hamilton-Brehm SD, Davenport KW, Podar M, et al (2010) Complete genome sequence of the cellulolytic thermophile Caldicellulosiruptor obsidiansis OB47T. J Bacteriol 192: 6099–6100. 20851897 10.1128/JB.00950-10 PMC2976464

[11] Kataeva IA, Yang SJ, Dam P, Poole FL, 2nd, Yin Y, et al (2009) Genome sequence of the anaerobic, thermophilic, and cellulolytic bacterium “Anaerocellum thermophilum” DSM 6725. J Bacteriol 191: 3760–3761. 19346307 10.1128/JB.00256-09 PMC2681903

[12] van de Werken HJ, Verhaart MR, VanFossen AL, Willquist K, Lewis DL, et al (2008) Hydrogenomics of the extremely thermophilic bacterium Caldicellulosiruptor saccharolyticus. Appl Environ Microbiol 74: 6720–6729. 18776029 10.1128/AEM.00968-08 PMC2576683

[13] Blumer-Schuette SE, Lewis DL, Kelly RM (2010) Phylogenetic, microbiological, and glycoside hydrolase diversities within the extremely thermophilic, plant biomass-degrading genus Caldicellulosiruptor. Appl Environ Microbiol 76: 8084–8092. 20971878 10.1128/AEM.01400-10 PMC3008241

[14] Dam P, Kataeva I, Yang SJ, Zhou F, Yin Y, et al (2011) Insights into plant biomass conversion from the genome of the anaerobic thermophilic bacterium Caldicellulosiruptor bescii DSM 6725. Nucleic Acids Res 39: 3240–3254. 21227922 10.1093/nar/gkq1281 PMC3082886

[15] Allers T, Mevarech M (2005) Archaeal genetics – the third way. Nat Rev Genet 6: 58–73. 15630422 10.1038/nrg1504

[16] Noll KM, Vargas M (1997) Recent advances in genetic analyses of hyperthermophilic archaea and bacteria. Arch Microbiol 168: 73–80. 9238098 10.1007/s002030050472

[17] Boeke JD, LaCroute F, Fink GR (1984) A positive selection for mutants lacking orotidine-5′-phosphate decarboxylase activity in yeast: 5-fluoro-orotic acid resistance. Mol Gen Genet 197: 345–346. 6394957 10.1007/BF00330984

[18] Broschard TH, Lebrun-Garcia A, Fuchs RP (1998) Mutagenic specificity of the food mutagen 2-amino-3-methylimidazo[4,5-f]quinoline in Escherichia coli using the yeast URA3 gene as a target. Carcinogenesis 19: 305–310. 9498281 10.1093/carcin/19.2.305

[19] Lipscomb GL, Stirrett K, Schut GJ, Yang F, Jenney FE Jr, et al (2011) Natural competence in the hyperthermophilic archaeon Pyrococcus furiosus facilitates genetic manipulation: construction of markerless deletions of genes encoding the two cytoplasmic hydrogenases. Appl Environ Microbiol 77: 2232–2238. 21317259 10.1128/AEM.02624-10 PMC3067412

[20] Lucas S, Toffin L, Zivanovic Y, Charlier D, Moussard H, et al (2002) Construction of a shuttle vector for, and spheroplast transformation of, the hyperthermophilic archaeon Pyrococcus abyssi. Appl Environ Microbiol 68: 5528–5536. 12406746 10.1128/AEM.68.11.5528-5536.2002 PMC129897

[21] Peck RF, DasSarma S, Krebs MP (2000) Homologous gene knockout in the archaeon Halobacterium salinarum with ura3 as a counterselectable marker. Mol Microbiol 35: 667–676. 10672188 10.1046/j.1365-2958.2000.01739.x

[22] Sato T, Fukui T, Atomi H, Imanaka T (2003) Targeted gene disruption by homologous recombination in the hyperthermophilic archaeon Thermococcus kodakaraensis KOD1. J Bacteriol 185: 210–220. 12486058 10.1128/JB.185.1.210-220.2003 PMC141832

[23] Sato T, Fukui T, Atomi H, Imanaka T (2005) Improved and versatile transformation system allowing multiple genetic manipulations of the hyperthermophilic archaeon Thermococcus kodakaraensis. Appl Environ Microbiol 71: 3889–3899. 16000802 10.1128/AEM.71.7.3889-3899.2005 PMC1169065

[24] Chung DH, Huddleston JR, Farkas J, Westpheling J (2011) Identification and characterization of CbeI, a novel thermostable restriction enzyme from Caldicellulosiruptor bescii DSM 6725 and a member of a new subfamily of HaeIII-like enzymes. J Ind Microbiol Biotechnol 38: 1867–1877. 21604181 10.1007/s10295-011-0976-x PMC4269323

[25] Gallagher LA, McKevitt M, Ramage ER, Manoil C (2008) Genetic dissection of the Francisella novicida restriction barrier. J Bacteriol 190: 7830–7837. 18835994 10.1128/JB.01188-08 PMC2583604

[26] Cue D, Lam H, Dillingham RL, Hanson RS, Flickinger MC (1997) Genetic manipulation of Bacillus methanolicus, a gram-positive, thermotolerant methylotroph. Appl Environ Microbiol 63: 1406–1420. 9097439 10.1128/aem.63.4.1406-1420.1997 PMC168436

[27] Purdy D, O'Keeffe TA, Elmore M, Herbert M, McLeod A, et al (2002) Conjugative transfer of clostridial shuttle vectors from Escherichia coli to Clostridium difficile through circumvention of the restriction barrier. Mol Microbiol 46: 439–452. 12406220 10.1046/j.1365-2958.2002.03134.x

[28] Jennert KC, Tardif C, Young DI, Young M (2000) Gene transfer to Clostridium cellulolyticum ATCC 35319. Microbiology 146 Pt 12: 3071–3080. 10.1099/00221287-146-12-3071 11101665

[29] Accetto T, Peterka M, Avgustin G (2005) Type II restriction modification systems of Prevotella bryantii TC1-1 and Prevotella ruminicola 23 strains and their effect on the efficiency of DNA introduction via electroporation. FEMS Microbiol Lett 247: 177–183. 15936894 10.1016/j.femsle.2005.05.016

[30] Donahue JP, Israel DA, Peek RM, Blaser MJ, Miller GG (2000) Overcoming the restriction barrier to plasmid transformation of Helicobacter pylori. Mol Microbiol 37: 1066–1074. 10972825 10.1046/j.1365-2958.2000.02036.x

[31] Chen CK, Boucle CM, Blaschek HP (1996) Factors involved in the transformation of previously non-transformable Clostridium perfringens type B. FEMS Microbiol Lett. 140: 185–191. 10.1111/j.1574-6968.1996.tb08334.x 8764481

[32] Guss A, Olson D, Caiazza N, Lynd L (2012) Dcm methylation is detrimental to plasmid transformation in *Clostridium thermocellum*. Biotechnol Biofuels in press. 10.1186/1754-6834-5-30 PMC3536630 22559230

[33] Reinisch KM, Chen L, Verdine GL, Lipscomb WN (1995) The crystal structure of HaeIII methyltransferase convalently complexed to DNA: an extrahelical cytosine and rearranged base pairing. Cell 82: 143–153. 7606780 10.1016/0092-8674(95)90060-8

[34] Ehrlich M, Gama-Sosa MA, Carreira LH, Ljungdahl LG, Kuo KC, et al (1985) DNA methylation in thermophilic bacteria: N4-methylcytosine, 5-methylcytosine, and N6-methyladenine. Nucleic Acids Res 13: 1399–1412. 4000939 10.1093/nar/13.4.1399 PMC341080

[35] Roberts RJ, Vincze T, Posfai J, Macelis D (2010) REBASE--a database for DNA restriction and modification: enzymes, genes and genomes. Nucleic Acids Res 38: D234–236. 19846593 10.1093/nar/gkp874 PMC2808884

[36] Mai V, Lorenz WW, Wiegel J (1997) Transformation of Thermoanaerobacterium sp. strain JW/SL-YS485 with plasmid pIKM1 conferring kanamycin resistance. FEMS Microbiol Lett 148: 163–167.

[37] Shaw AJ, Hogsett DA, Lynd LR (2009) Identification of the [FeFe]-hydrogenase responsible for hydrogen generation in Thermoanaerobacterium saccharolyticum and demonstration of increased ethanol yield via hydrogenase knockout. J Bacteriol 191: 6457–6464. 19648238 10.1128/JB.00497-09 PMC2753037

[38] Cannio R, Contursi P, Rossi M, Bartolucci S (2001) Thermoadaptation of a mesophilic hygromycin B phosphotransferase by directed evolution in hyperthermophilic Archaea: selection of a stable genetic marker for DNA transfer into Sulfolobus solfataricus. Extremophiles 5: 153–159. 11453458 10.1007/s007920100189

[39] Tyurin MV, Desai SG, Lynd LR (2004) Electrotransformation of Clostridium thermocellum. Appl Environ Microbiol 70: 883–890. 14766568 10.1128/AEM.70.2.883-890.2004 PMC348934

[40] Peng H, Fu B, Mao Z, Shao W (2006) Electrotransformation of Thermoanaerobacter ethanolicus JW200. Biotechnol Lett 28: 1913–1917. 16988780 10.1007/s10529-006-9184-6

[41] Karnik SS, Gopinathan KP (1983) Transfection of Mycobacterium smegmatis SN2 with mycobacteriophage I3 DNA. Arch Microbiol 136: 275–280. 6667087 10.1007/BF00425216

[42] Shaw AJ, Hogsett DA, Lynd LR (2010) Natural competence in Thermoanaerobacter and Thermoanaerobacterium species. Appl Environ Microbiol 76: 4713–4719. 20472726 10.1128/AEM.00402-10 PMC2901744

[43] Peteranderl R, Canganella F, Holzenburg A, Wiegel J (1993) Induction and Regeneration of Autoplasts from Clostridium thermohydrosulfuricum JW102 and Thermoanaerobacter ethanolicus JW200. Appl Environ Microbiol 59: 3498–3501. 16349076 10.1128/aem.59.10.3498-3501.1993 PMC182483

[44] Trieu-Cuot P, Carlier C, Martin P, Courvalin P (1987) Plasmid transfer by conjugation from Escherichia coli to Gram-positive bacteria. FEMS Microbiology Letters 48: 289–294.

[45] Grogan DW (2003) Cytosine methylation by the SuaI restriction-modification system: implications for genetic fidelity in a hyperthermophilic archaeon. J Bacteriol 185: 4657–4661. 12867480 10.1128/JB.185.15.4657-4661.2003 PMC165766

[46] Benson DA, Karsch-Mizrachi I, Lipman DJ, Ostell J, Sayers EW (2010) GenBank. Nucleic Acids Res 38: D46–51. 19910366 10.1093/nar/gkp1024 PMC2808980

[47] Rao BS, Buckler-White A (1998) Direct visualization of site-specific and strand-specific DNA methylation patterns in automated DNA sequencing data. Nucleic Acids Res 26: 2505–2507. 9580708 10.1093/nar/26.10.2505 PMC147555

[48] Bart A, van Passel MW, van Amsterdam K, van der Ende A (2005) Direct detection of methylation in genomic DNA. Nucleic Acids Res 33: e124. 16091626 10.1093/nar/gni121 PMC1184226

[49] Arber W, Dussoix D (1962) Host specificity of DNA produced by Escherichia coli. I. Host controlled modification of bacteriophage lambda. J Mol Biol 5: 18–36. 13862047 10.1016/s0022-2836(62)80058-8

[50] Malone T, Blumenthal RM, Cheng X (1995) Structure-guided analysis reveals nine sequence motifs conserved among DNA amino-methyltransferases, and suggests a catalytic mechanism for these enzymes. J Mol Biol 253: 618–632. 7473738 10.1006/jmbi.1995.0577

[51] Timinskas A, Butkus V, Janulaitis A (1995) Sequence motifs characteristic for DNA [cytosine-N4] and DNA [adenine-N6] methyltransferases. Classification of all DNA methyltransferases. Gene 157: 3–11. 7607512 10.1016/0378-1119(94)00783-o

[52] Klimasauskas S, Timinskas A, Menkevicius S, Butkiene D, Butkus V, et al (1989) Sequence motifs characteristic of DNA[cytosine-N4]methyltransferases: similarity to adenine and cytosine-C5 DNA-methylases. Nucleic Acids Res 17: 9823–9832. 2690010 10.1093/nar/17.23.9823 PMC335216

[53] Matveyev AV, Young KT, Meng A, Elhai J (2001) DNA methyltransferases of the cyanobacterium Anabaena PCC 7120. Nucleic Acids Res 29: 1491–1506. 11266551 10.1093/nar/29.7.1491 PMC31280

[54] Kawarabayasi Y, Sawada M, Horikawa H, Haikawa Y, Hino Y, et al (1998) Complete sequence and gene organization of the genome of a hyper-thermophilic archaebacterium, Pyrococcus horikoshii OT3 (supplement). DNA Res 5: 147–155. 9679203 10.1093/dnares/5.2.147

[55] Cohen HM, Tawfik DS, Griffiths AD (2002) Promiscuous methylation of non-canonical DNA sites by HaeIII methyltransferase. Nucleic Acids Res 30: 3880–3885. 12202773 10.1093/nar/gkf507 PMC137429

[56] Hethke C, Geerling AC, Hausner W, de Vos WM, Thomm M (1996) A cell-free transcription system for the hyperthermophilic archaeon Pyrococcus furiosus. Nucleic Acids Res 24: 2369–2376. 8710509 10.1093/nar/24.12.2369 PMC145958

[57] Sedmak JJ, Grossberg SE (1977) A rapid, sensitive, and versatile assay for protein using Coomassie brilliant blue G250. Anal Biochem 79: 544–552. 68686 10.1016/0003-2697(77)90428-6

[58] Crowe ML (2005) SeqDoC: rapid SNP and mutation detection by direct comparison of DNA sequence chromatograms. BMC Bioinformatics 6: 133. 15927052 10.1186/1471-2105-6-133 PMC1156871

